# Medical physicist should be a planner in the treatment planning process

**DOI:** 10.1002/acm2.70185

**Published:** 2025-07-15

**Authors:** Dongxu Wang, Douglas E. Prah, Yi Rong

**Affiliations:** ^1^ Department of Medical Physics Memorial Sloan Kettering Cancer Center New York USA; ^2^ Department of Radiation Oncology Medical College of Wisconsin Milwaukee Wisconsin USA; ^3^ Department of Radiation Oncology Mayo Clinic Arizona Phoenix Arizona USA

**Keywords:** medical dosimetrists, qualified medical physicists, radiotherapy treatment planner

## 1 INTRODUCTION

In the United States, both medical physicists and medical dosimetrists play essential roles in radiotherapy. Since its inception, the profession of medical dosimetry has focused primarily on the creation of treatment plans and related clinical tasks. In contrast, the involvement of medical physicists in the treatment planning process has been more variable and continues to evolve as clinical practices and professional expectations shift. Traditionally, the most common model of collaboration between dosimetrists and physicists involves physicists serving as secondary reviewers, ensuring quality and safety after the dosimetrist has completed the initial treatment plan. However, with the increasing complexity of modern radiotherapy techniques, which demand greater precision, personalization, and interdisciplinary coordination, many institutions face growing challenges in recruiting experienced treatment planners capable of handling complex cases. The need for extensive on‐the‐job training, often provided by senior dosimetrists and occasionally by physicists, places additional strain on departments already affected by workforce shortages. This situation raises an important question: should medical physicists assume a more formalized role as treatment planners? This debate examines the proposition that clinical medical physicists should participate routinely in the treatment planning process, not merely as reviewers or technical advisors, but as active contributors working alongside dosimetrists and radiation oncologists. The faculty physicist arguing for the proposition is Dr. Dongxu Wang from Memorial Sloan Kettering Cancer Center, while the faculty physicist arguing against the proposition is Dr. Douglas Prah from the Medical College of Wisconsin.

Dongxu Wang, PhD, MBA, received his PhD in Medical Physics from the University of Wisconsin‐Madison in 2011. After graduate school, he joined the University of Iowa Hospitals and Clinics as a faculty physicist. While at the University of Iowa, he studied part‐time and received his master's degree in business administration (MBA) in 2019. He is now an Associate Attending Physicist at Memorial Sloan Kettering Cancer Center. Dr. Wang's earlier expertise and focus were in proton therapy and proton imaging. Lately he is active in advancing medical physics leadership and professionalism education using the case study method.

Douglas Prah, PhD, DABR, is a board‐certified medical physicist, Associate Professor of Radiation Oncology, and the Director of Advanced Care & Technology at Froedtert & the Medical College of Wisconsin. He earned his PhD in Biophysics from the Medical College of Wisconsin and specializes in radiation beam modeling, treatment planning, and integrating advanced technologies into clinical workflows. Dr. Prah chairs the Service and Technology Implementation and Review Committee and oversees medical dosimetry services across the enterprise. He is also an APEx Surveyor and serves on the Practice Accreditation Subcommittee for the American Society for Radiation Oncology, where he supports national efforts to improve quality and safety in radiation oncology. Dr. Prah is committed to advancing the field through collaborative leadership, innovation, and education.

## 2 OPENING STATEMENT

### For the proposition: Dongxu Wang, PhD, MBA

Clinical medical physicists in radiation oncology often perform treatment planning for special procedures such as brachytherapy, stereotactic radiosurgery (SRS), stereotactic body radiotherapy (SBRT), and online adaptive therapy. There are three different levels of involvement for medical physicists (MP) in the process of treatment planning, which includes (1). MP not responsible for treatment plan quality; (2). MP reviews treatment plan quality; and (3). MP directly participates in treatment planning. For most of the radiation oncology departments, MPs usually do not perform routine treatment planning for external beam radiation therapy, except in hospitals under dosimetrist staffing constraints or from a historical arrangement. I propose, however, clinical therapy physicists should broadly incorporate treatment planning into their regular job duties for the following reasons.

### The professional reason

Both physicists and dosimetrists bring valuable, complementary skills to the treatment planning process. With physics knowledge, qualified medical physicists (QMP) manage machine limitations or dosimetric uncertainties effectively in treatment planning. With the advancement of knowledge‐based auto‐planning, the technical considerations and technical constraints play a more important role in achieving optimal treatment planning, which often requires a balance between deliverability and dose distribution.

One may argue that, if the physicist has adequately modeled the beam in the treatment planning system (TPS), no additional consideration is needed. This view is idealistic. As clinical physicists, we know that even the best beam model or TPS still has its limitations, especially for highly modulated fields. Also, certain practical knowledge is necessary in the modern planning process, such as knowledge of image guidance uncertainty, motion management technology, and so forth. Consequently, this knowledge leads to the decisions about the proper target volume margin, image guidance protocols, gantry clearance, and so forth. On these technical matters, the physics aspects should be integrated into a planner's knowledge. Many dosimetrists have successfully attained such physics knowledge for practical purposes. As a profession, based on their training, physicists are naturally suited for such an integrated planner role, without removing the need for an independent plan check by another QMP.

### The operational process reason

In the role of a QMP planner, special physics consults can be integrated into treatment planning. Special physics consults, such as complex multi‐modality image registration, prior radiation dose accumulation, motion assessment, special dosimetry, and so forth, are necessary parts of the treatment planning process and should be arranged as such. This integration streamlines the workflow for patients, removing the need for different people to perform different tasks during treatment planning.

**FIGURE 1:**
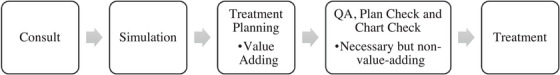
Value stream mapping of typical external beam radiotherapy workflow for a patient. The QA, Plan Check and Chart Check are necessary but non‐value‐adding.

Using the “Lean” process method of value stream mapping, the workflow for a typical radiation therapy patient is illustrated in Figure 1.

Here, the integrated treatment planning is a *value‐adding process*, the most important of a Lean process. “Treatment planning” here broadly includes the special physics consult as mentioned earlier, as well as the necessary physics knowledge in the planner's role. Quality assurance (QA) and plan check, however, are *necessary but non‐value‐adding* processes in the Lean concept. While quality control and radiation safety must not be downplayed by any means, the routine involvement in the value‐adding process of each patient's care adds to the planner's role's professional vitality and visibility. It should be noted that QA, Plan Check and Chart Check, while routine in radiation oncology practice, are not universal, or even common, practices in other medical specialties. Hospitals, administrators, or other stakeholders may not fully appreciate the *value* of medical physicists unless we participate more in the *value‐adding process*.

Finally, along the line of business justification, dosimetry treatment planning has direct billing codes in the United States, such as 3D conformal plan (CPT 77295) and IMRT plan (CPT 77301). None of the QA or plan check work has billing codes directly attributed to it, even though the underlying work effort is reflected in certain other billing codes. In many hospital administrations’ business frameworks, participating in or even leading billable procedures is a manifestation of the profession's value. Medical physicists should routinely participate in treatment planning, a value‐adding process, especially considering that physicists are well‐trained and fully qualified for this job.

### Against the proposition: Douglas E. Prah, PhD

Medical dosimetrists are invaluable and essential members of the radiation oncology team, responsible for generating a continuous stream of high‐quality treatment plans based on often demanding written treatment planning directives. Their efficiency and skill are unmatched. Board‐certified by the American Association of Medical Dosimetrists (AAMD), they maintain their certification through ongoing medical education. The purpose of this essay is not to undermine their critical role but to offer a support system that enhances their contributions to the radiation oncology team.

The opening statement is simple, medical physicists must be expert dosimetrists. Medical physicists are responsible for the safety and quality of treatment plans, and as such they must be comprehensive experts of treatment planning techniques and the TPS, including the backend software, the machine models and dose calculation algorithms, the frontend software, the functionality and operation of the software, and the output, the treatment plans and documentation. They must not only be generally knowledgeable of the TPS backend and output, they must be expert planners themselves, this includes the frontend of the TPS and the planning techniques to generate high quality treatment plans. Like the relationship between a coach and an athlete, it does not imply that physicists should be as efficient as dosimetrists or that a physicist should replace a dosimetrist for daily responsibilities, only that they are comprehensive experts of the treatment planning and are able to give evaluation, advice, and training. In radiation oncology departments, physics and dosimetry work together as a team to maintain a high level of quality treatment plans. To enhance this relationship and improve the quality of treatment plans, a physicist must be a comprehensive TPS expert.

### Collaboration

In radiation oncology, treatment planning is a collaborative effort between physicians, dosimetrists, and physicists. Physics often supervises their dosimetry counterparts. Physicists should be capable of performing the duties of the other team members. A physicist with dosimetry expertise can actively contribute to this team. Furthermore, with expertise in dosimetry, the physicist provides improved education to the dosimetry team.

### Beyond good

Voltaire, a French writer and philosopher, paraphrasing an Italian proverb, stated, “*Le mieux est l'ennemi du bien*,” which translates to “The best is the enemy of the good.”^1^ When a challenging treatment planning case consumes copious amounts of time and effort, it is easier to be convinced that enough has been done to improve a treatment plan that is already “good.” However, what if more could have been done, or even worse, an accidental mistake was introduced into the plan, resulting in suboptimal dosimetry. If physics does not have a comprehensive expert understanding of treatment planning techniques and the TPS, they must rely on the judgment of the dosimetrist and assume flawless implementation. This is more problematic in clinics for which there might not be an opportunity to compare varying plan quality across multiple dosimetrists. In the case of systematic methods that result in suboptimal plan quality, the physicist without expert planning skills would not be able to develop an appreciation of a true high‐quality plan for a specific disease site. For example, during optimization, if an incorrect setting or property was accidentally used on an optimization constraint, this may result in suboptimal target or organ coverage. With incorrect settings in place, a dosimetrist may even compensate by modifying other constraints or settings. Without an expert understanding of the treatment planning techniques and systems, physics would be unlikely to differentiate the upstream setting that impacted the dose distribution. Ultimately physics would fail in their responsibility of maintaining the quality and safety of the treatment plan. Physicists must be expert dosimetrists to identify accidental mistakes made during planning; without this expertise, we can only provide a qualitative assessment based on empirically derived past experiences.

### Principal investigator

Radiation oncology is a highly technical field for which issues may arise during treatment planning or delivery. These technical issues must be investigated by the medical physicist. To perform the investigational analysis of a suspected incident, the physics team must be comprehensive experts of treatment planning techniques and the TPS to reproduce the incident.

### Driving innovation in radiation oncology

Research in radiation oncology constantly propels the field forward. As new techniques or methods are introduced into the clinic, each must first be understood, implemented, and evaluated by the team. While dosimetry is tasked with generating treatment plans, they often lack the time or resources to assist with the technical complexities of integrating new technologies or treatment methods. As a result, dosimetry relies heavily on physics for guidance in the implementation process. Without the in‐depth expertise gained through hands‐on experience with new technologies in the TPS, physics would lack the foundational knowledge to support the treatment plans developed by dosimetrists.

## 3 REBUTTAL

### For the proposition: Dongxu Wang, PhD, MBA

Dr. Prah's opening statement did not directly argue against the debate proposition. Dr. Prah stated that “medical physicists must be expert dosimetrists,” and physicists are “comprehensive experts of the treatment planning.” We agree on these points but differ in the scope of the debate proposition: I believe physicists should actively participate in the treatment planning process, while Dr. Prah suggests that physicists should be experts of but not participants in the treatment planning process. Being an expert without participating presents a challenging distinction with slim feasibility.

Dr. Prah offered a few explanations. He first argued that “medical physicists must be expert dosimetrists,” but that “does not imply that physicists should be as efficient as dosimetrists.” In my experience, expertise and efficiency go hand in hand. The daily challenge of treatment planning often involves optimizing a plan within a limited timeframe. Without efficiency, theoretical improvements in plan quality cannot be achieved in a practical setting. In a common plan check situation, a physicist believes a plan's quality can be further improved, but the dosimetrist does not see an easy approach. The physicist needs to prove their hypothesis by suggesting certain optimization objectives, cost function weights, and optimization structure and, most crucially, have the plan further optimized and fully checked before the patient's scheduled start time. A physicist must be efficient to excel in treatment planning, not just through repeated trial and error without time constraint.

Dr. Prah also compared the relationship between “a coach and an athlete” to that of a physicist and dosimetrist. This analogy appears to suggest that a physicist is like a good coach, an expert in a sport but not necessarily playing well in it. Most of the coaches were once good athletes too. A physicist must have sufficient experience in treatment planning to be able to coach other team members effectively. Dr. Prah stated that physicists are “comprehensive experts of the treatment planning.” To get there, a physicist needs to do plenty of treatment planning. Physicists’ participatory experience in treatment planning is necessary for them to attain the level of competency to “give evaluation, advice, and training,” as cited by Dr. Prah. While not all physicists must be performing treatment planning all the time, all physicists should have done treatment planning at some point in their career after the training.

I agree with Dr. Prah and recognize the many other important roles played by physicists, including implementing new technologies and ensuring safety and quality. These important duties demand effort and attention from the physicists. A physicist cannot be a full‐time planner, nor can physicists replace dosimetrists in all situations or institutions. My proposition is that more physicists should participate in treatment planning, in parallel with dosimetrists, instead of solely being plan checkers. This proposal can be achieved by revisiting the treatment planning workflow and incorporating the physicist's partial role as a planner. The physicist's planning workload may be time‐based or case‐based, and individual effort may differ by the physicist and by the clinic. As laid out in the opening statements and for various good reasons, for physicists to deliver the best in treatment planning, participation is key.

### Against the proposition: Douglas E. Prah, PhD

The difference in position is slight, but an essential distinction in arguments must be made. The medical physicist has many routine responsibilities within the department. Assigning additional routine responsibilities to the medical physicist may create an undue burden. As was previously stated, the medical physicist should be an expert planner. If time permits, the medical physicist may participate in the treatment planning process, which aids in maintaining the necessary skills to be a comprehensive TPS expert, including generating high‐quality treatment plans. However, where this may pose an undue burden to dosimetry is routine treatment planning assignments. For a department in which medical physics would be tolerant of routine treatment planning assignments, it would require a budgeted full‐time equivalent effort to be allocated to the task of treatment planning. If dedicated financial support is not in place, a strained department may begin to rely on physicists to supplement the dosimetrists’ efforts. Medical physicists are often in salaried positions and often generously offer their time to support the clinic. However, without proper medical physics staffing levels, fulfilling all their other obligations to the department, including additional routine treatment plan generation, would lead to employee burnout. Furthermore, the medical physicist should not become a staffing tool to bridge dosimetry staffing insufficiency. This is even more true during the current staffing shortages seen in the US within radiation oncology. The department must ensure that proper staffing of both dosimetry and medical physics is provided. In an ideal setting, the department could budget a small fraction of time for the medical physicist to work on treatment plans, but they should not be drawn into backup treatment planning support for a short‐staffed dosimetry section.

Given the higher salary of the medical physics staff, regardless of the revenue generated from treatment planning billing codes, the more cost‐effective solution is to allocate these tasks exclusively to dosimetrists. However, if the purpose of these tasks is solely to maintain the skills for treatment planning expertise, this additional effort may be worth the extra expense. Regardless, routinely relying on medical physics for this responsibility would not be cost‐effective. Participation in the treatment planning process should be managed to provide support to the dosimetrist that maximizes the dosimetrist's productivity. Medical physics should be allowed to participate in the treatment planning process but not be routinely relied on for plan generation.

Medical physicists in radiation oncology are pushed and pulled in many directions in the department. Their expertise is used to maintain quality assurance standards across an array of equipment and processes in the department. This expertise is of the utmost importance to the department. Other disciplines do not check the medical physicists’ work. Consequently, this work must not be compromised. Assigning routine treatment planning tasks to the medical physicist creates the potential to undermine quality assurance in radiation oncology. With the proper management and staffing levels, medical physicists may participate in the treatment planning and even sharpen their skills as a comprehensive TPS expert, but that effort must never compromise their primary responsibilities to the department.

## CONFLICT OF INTEREST STATEMENT

Dongxu Wang reports receiving an honorarium from Mevion Medical Systems, Inc., and a licensing fee payment from Ion Beam Applications, S.A.; neither is related to this work.

## References

[1] Radcliffe S . Concise Oxford Dictionary of Quotations. 5th ed. Oxford University Press; 2006.

